# Pomegranate Peel Polyphenol Extract Ameliorates Hyperuricemia by Inhibiting Uric Acid Synthesis and Reabsorption

**DOI:** 10.1111/cbdd.70376

**Published:** 2026-08-06

**Authors:** Zeyu Yin, Shufei Liang, Gangao Yang, Pingping Guo, Chao Wang, Qingqing Han, Xinhua Song

**Affiliations:** ^1^ School of Life Sciences and Medicine Shandong University of Technology Zibo Shandong People's Republic of China; ^2^ Innovation Institute of Integrated Traditional Chinese and Western Medicine Shandong First Medical University & Shandong Academy of Medical Sciences Jinan Shandong Province China

**Keywords:** ellagic acid, hyperuricemia, network pharmacology, PI3K‐AKT pathway, pomegranate peel polyphenol extract

## Abstract

Hyperuricemia (HUA) is a common metabolic disorder with limited safe and effective therapeutic options. This study integrated GEO dataset mining and network pharmacology to explore the anti‐HUA efficacy and mechanism of pomegranate peel polyphenol extract (PPE). In a mouse model of HUA induced by potassium oxonate and 5% fructose water, PPE significantly reduced serum, urinary, and fecal uric acid levels, attenuated the increases in creatinine and blood urea nitrogen, improved estimated glomerular filtration rate, and ameliorated renal pathological damage, inflammation, and xanthine oxidase activity. Integration of GEO‐derived HUA‐related genes and computational target prediction for PPE identified 44 common targets, and protein–protein interaction network analysis revealed core targets including Akt1. KEGG enrichment highlighted the PI3K‐AKT signaling pathway as a key mediator. Western blotting in vivo and in vitro confirmed that PPE suppressed PI3K‐AKT phosphorylation and downregulated the urate reabsorption transporters URAT1 and GLUT9. Furthermore, experiments in UA‐induced HK‐2 cells demonstrated that ellagic acid, a major bioactive component of PPE, acted through the same pathway. These findings indicate that PPE reduces uric acid levels and protects the kidney via modulation of the PI3K‐AKT pathway, providing an integrative data‐driven rationale for its potential as a functional food or pharmaceutical agent against HUA.

## Graphical abstract

The graphical abstract illustrates the action mechanisms through which PPE alleviates HUA by inhibiting XOD activity, and by suppressing the PI3K‐AKT signaling pathway to downregulate the expression of the urate reabsorption transporters URAT1 and GLUT9, thereby promoting renal UA excretion and attenuating renal injury.

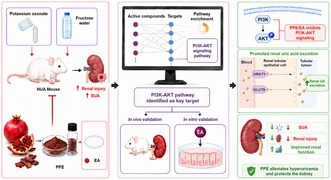

## 1 Introduction

Hyperuricemia (HUA) is a metabolic disorder characterized by elevated serum uric acid (SUA) levels exceeding the normal saturated threshold, a condition arising from purine metabolism dysfunction (Hou et al. 2021; C. Zhang et al. 2020). The clinical diagnostic criteria are defined as SUA levels > 420 μmol/L in men and > 360 μmol/L in women on two separate fasting occasions (Johnson et al. 2026). In recent years, HUA has emerged as the “fourth highest” metabolic disorder following hypertension, hyperlipidemia, and hyperglycemia, with a persistently rising global prevalence and high comorbidity with the three classic metabolic diseases, jointly constituting an important risk factor for cardiovascular and metabolic disorders (FitzGerald et al. 2020; Huang et al. 2025; C. Zhang et al. 2020).

The pathogenesis of HUA is closely associated with increased UA production and/or decreased UA excretion (B. Yang et al. 2023). Approximately 20% of body uric acid (UA) is derived from the intake of high‐purine foods, while the remaining 80% originates from endogenous purine metabolism. Xanthine oxidase (XOD) is the rate‐limiting enzyme in purine metabolism, catalyzing the conversion of hypoxanthine to xanthine and subsequently to UA. UA production can be significantly increased under conditions of enhanced XOD activity, the rate‐limiting enzyme in purine metabolism (Y. Zhang, Zhu, et al. 2024). The kidney is the primary organ responsible for UA excretion, accounting for approximately two‐thirds of free UA elimination. Renal UA excretion is largely determined by reabsorption processes in proximal tubules (Guo et al. 2025). Approximately 90% of filtered UA is reabsorbed, a process mediated by two key transporters with distinct subcellular localization and functional division. URAT1 is localized on the apical (luminal) membrane of proximal tubular epithelial cells and responsible for transporting UA from the tubular lumen into the cytoplasm. GLUT9 is expressed on the basolateral membrane and mediates the efflux of UA from tubular epithelial cells into the renal interstitium and systemic circulation. The coordinated action of the two transporters completes transepithelial UA reabsorption and plays a critical regulatory role in maintaining UA homeostasis (Guo et al. 2025; Matsushita et al. 2025; C. Wu et al. 2025; Y. Zhang et al. 2021). Dysregulation of these transporters impairs renal uric acid excretion and thus contributes to the development of HUA (Guo et al. 2025; B. Yang et al. 2023). Beyond renal elimination, the intestine serves as a major extra‐renal pathway for UA clearance, accounting for approximately one‐third of total UA excretion in humans. This process is coordinately regulated by intestinal epithelial transporters and gut microbiota. Notably, gut microbiota metabolic reprogramming has been widely recognized as a core driver of host metabolic disorders; it disrupts systemic purine and UA homeostasis, and further aggravates renal injury through the gut‐kidney axis (Wang et al. 2026). Given that natural polyphenols in PPE undergo extensive biotransformation by gut microbiota in vivo, the intestinal pathway likely represents an important non‐absorptive mechanism underlying the anti‐hyperuricemic effects of PPE. In turn, persistent HUA itself induces progressive renal injury and inflammation. Renal urate crystal deposition triggers oxidative stress and inflammatory cascades, eventually leading to tubulointerstitial fibrosis, elevated serum creatinine and blood urea nitrogen levels, and increased secretion of pro‐inflammatory cytokines including IL‐1β, IL‐6, and TNF‐α (Xuan et al. 2026; Yang, Ying, et al. 2025). Accordingly, timely intervention is essential for preserving renal function, alleviating inflammatory damage, and preventing disease complications.

Currently, pharmacological interventions remain the primary approach for HUA treatment, which are mainly categorized into three classes—XOD inhibitors (allopurinol and febuxostat), uricosuric agents (benzbromarone and probenecid), and uricase formulations (rasburicase and pegloticase) (Huang et al. 2025). However, each class presents significant limitations. Allopurinol is prone to causing severe cutaneous adverse reactions and requires dose adjustment in patients with renal insufficiency (Y. Bai et al. 2023; Huang et al. 2025). Although febuxostat exhibits stronger inhibitory effects and higher specificity, it carries a risk of hepatotoxicity (Deng et al. 2024; Huang et al. 2025). Benzbromarone has not been approved in the United States and has been withdrawn from most European markets due to severe hepatotoxicity, while probenecid is associated with poor efficacy and drug–drug interactions (Huang et al. 2025). Given the room for improvement in the safety and long‐term tolerability of current HUA treatments, exploring novel intervention strategies with favorable profiles is of great clinical significance (Huang et al. 2025; B. Yang et al. 2022; L. Yang, Liu, et al. 2025). Traditional Chinese medicine (TCM), characterized by its multi‐target and multi‐pathway mechanisms, has emerged as an important complementary and potential alternative strategy for HUA treatment (Y. Li et al. 2025; L. Yang, Liu, et al. 2025). Studies have demonstrated that single herbs such as *Astragali radix* and *Plantaginis semen*, along with their active constituents including flavonoids and saponins, exert dual regulatory effects by inhibiting UA synthesis and promoting UA excretion (Y. Li et al. 2025; Y. Zhang, Zhu, et al. 2024). Classical formulas like Simiao San achieve synergistic effects through multiple components, simultaneously regulating UA metabolism and alleviating inflammation (Y. Li et al. 2025; Zhou et al. 2026). Mechanistically, beyond modulating urate metabolic pathways, TCM can inhibit inflammatory pathways such as NF‐κB, thereby reducing oxidative stress and tissue damage (T. Wang, Li, et al. 2025; B. Yang et al. 2023; J. Yang, Xu, et al. 2025). Moreover, TCM exhibits mild side effects and favorable tolerability, making it suitable for long‐term intervention (Y. Li et al. 2025; L. Yang, Liu, et al. 2025). In recent years, research on bioactive components from food and medicine homology resources has become a research hotspot in the field of metabolic disease intervention, with dual‐pathway action and multi‐target characteristics being the core advantages of such components (M.‐Y. Li et al. 2026). As representative bioactive substances of food and medicine homology, natural polyphenols typically exert therapeutic effects on metabolic diseases through two complementary pathways, the blood absorptive pathway and the non‐absorptive pathway. The former allows active ingredients to enter the circulation and act directly on target organs, while the latter enables components to regulate gut microbiota, intestinal barrier function and local immunity without entering the systemic circulation (Zhai et al. 2026). However, current research on TCM for HUA treatment still faces significant limitations (H. Bai et al. 2024). The active components and therapeutic targets of many herbal medicines remain unclear, and the lack of systematic study design has prevented the establishment of standardized protocols for mechanism elucidation and efficacy evaluation, thereby hindering clinical translation. Therefore, conducting more systematic and in‐depth investigations into TCM for the management of primary HUA is of great importance for the development of safe and effective intervention strategies.

Pomegranate peel is a characteristic medicinal material resource in Shandong Province with prominent regional industrial advantages (Maphetu et al. 2022). It is rich in hydrolyzable tannins (such as punicalagin and ellagic acid), flavonoids, and other active components (J. Du et al. 2025; Singh et al. 2023). In traditional Chinese medicine, pomegranate peel is documented to exert effects of astringing the intestine to stop diarrhea, hemostasis, and expelling parasites, and has a long history of adjuvant application for intestinal disorders and inflammatory conditions (Maphetu et al. 2022; Singh et al. 2023). Modern pharmacological studies have demonstrated that PPE possesses a variety of biological activities, including antioxidant, antibacterial, anti‐inflammatory, anticancer (mechanistically involving the regulation of key post‐translational modifications such as SUMOylation (Y. Wu et al. 2026)), neuroprotective, and immunomodulatory effects (J. Du et al. 2025; González‐González et al. 2024; H. Liu, Li, et al. 2024; H. Liu, Yan, et al. 2024; S. Liu, Dong, et al. 2024). Previous studies have confirmed that punicalagin (PU), the core active component of PPE, can down‐regulate the expression of urate reabsorption proteins and up‐regulate the expression of urate excretion proteins in the kidney, inhibit the activation of inflammatory pathways, and ameliorate glycolipid metabolism disorders (Han et al. 2025; L. Yang, Liu, et al. 2025). Studies have shown that PU is first converted into punicalin under the action of lactonohydrolase in vivo, and then further hydrolyzed into free ellagic acid (EA) by ester bond hydrolase (Z. Chen et al. 2026; Ramlagan et al. 2022). Compared with the macromolecule PU, which is easily hydrolyzed in the acidic environment of the body, identifying the direct targets of EA, a small molecule with simple structure and high stability, holds greater practical significance (J. Chen et al. 2023; Kmail 2026). However, research on the effects and mechanisms of PPE in improving HUA remains insufficient. Nevertheless, its good safety profile and multi‐target advantages provide a new strategy for the prevention and treatment of HUA and lay a foundation for the high‐value development of pomegranate peel resources. Traditional network pharmacology mostly relies on database‐predicted disease targets, which suffers from limitations such as high false positive rate and weak correlation with actual disease expression profiles. The integration of the GEO gene expression dataset allows screening of differentially expressed genes under real pathological conditions, which can be intersected with component target prediction results to effectively improve the accuracy of target screening and make the predicted pathways more consistent with the actual pathological process.

In the present study, the main active components (PU and EA) in PPE were quantitatively analyzed by UFLC‐MS/MS. Subsequently, the UA‐lowering efficacy and renoprotective effects of PPE were systematically evaluated using a HUA mouse model and a UA‐induced human renal proximal tubular epithelial cell (HK‐2) model. Network pharmacology combined with GEO chip dataset analysis was employed to predict its potential target mechanisms in alleviating HUA. Furthermore, Western blotting assays were conducted to verify the regulatory effect of PPE and its core active component EA on the PI3K‐AKT pathway and urate transporters both in vivo and in vitro. This study aims to clarify the dual mechanism of PPE in inhibiting UA synthesis and promoting UA excretion, reveal the key role of the PI3K‐AKT signaling pathway in its renal urate excretion regulation, and provide a scientific basis for the application of PPE in the treatment of HUA, as well as a theoretical foundation for its development as a functional food or pharmaceutical agent.

## 2 Materials and Methods

### 2.1 Reagents

Pomegranate peels were obtained from Beijing Tongrentang Zibo Drugstore Co. Ltd. (Zibo, China). Purified water was provided by Hangzhou Wahaha Group Co. Ltd. (Hangzhou, China). EA standard (purity ≥ 98%) was purchased from Macklin Biochemical Co. Ltd. (Shanghai, China). PU standard (purity ≥ 98%) was purchased from Yuanye Biotechnology Co. Ltd. (Shanghai, China). Methanol and acetonitrile (HPLC grade) were purchased from Fisher Chemical (Waltham, MA, USA). Formic acid was obtained from Fluka Analytical (Buchs, Switzerland). Dimethyl sulfoxide (DMSO, HPLC grade, ≥ 99.9%) was purchased from Shanghai Macklin Biochemical Co. Ltd. (Shanghai, China). Potassium oxonate (PO) (98%) was purchased from Shanghai Yuanye Biotechnology Co. Ltd. (Shanghai, China). Benzbromarone (≥ 98%), D‐fructose (99%), penicillin (sterile), streptomycin (sterile), UA (≥ 98%), glycine (> 99.00%), TBST, hematoxylin and eosin (H&E) staining kit, and protein marker (15–130 kDa) were purchased from Beijing Solarbio Science & Technology Co. Ltd. (Beijing, China). UA assay kits, creatinine (CRE) assay kits, blood urea nitrogen (BUN) assay kits, and XOD assay kits were obtained from Nanjing Jiancheng Technology Co. Ltd. (Nanjing, China). The antibodies used for Western blotting, including β‐Actin Rabbit mAb, SLC2A9 Rabbit pAb, URAT1 Rabbit mAb, IL‐6 Rabbit pAb, TNF‐α Rabbit pAb, PI3K Rabbit mAb, Phospho‐PI3K Rabbit pAb, Phospho‐AKT1 Rabbit mAb, and AKT1 Rabbit mAb, were all purchased from Beijing Bioss Biotechnology Co. Ltd. (Beijing, China).

### 2.2 Preparation of Pomegranate Peel Polyphenol Extract

Dried pomegranate peels were pulverized using a grinder and passed through a 60‐mesh sieve. Accurately weighed 50 g of the sieved powder was wrapped in filter paper to prepare a medicinal bag. An appropriate amount of petroleum ether (approximately one‐third of the extraction tank volume) was added to a Soxhlet extractor, and the mixture was heated under reflux at 50°C–60°C until the liquid in the extraction tank became colorless and transparent, thereby removing the lipid components from the pomegranate peel powder. The medicinal bag was taken out, dried to remove residual petroleum ether, placed in a beaker, and 500 mL of 75% ethanol‐water solution was added. The beaker was sealed and allowed to stand overnight at room temperature. The medicinal bag and the ethanol‐water solution were then transferred together into the Soxhlet extractor and extracted under reflux at 50°C–60°C for 12 h until the extract became colorless and transparent. The extract was collected and recovered. The extract was concentrated under reduced pressure at 50°C–60°C using a rotary evaporator, and the concentrate was dried under vacuum to obtain crude pomegranate peel polyphenol extract powder.

The crude powder was dissolved in 75% ethanol, and petroleum ether was added at a volume ratio of 1:1 (ethanol solution: petroleum ether). The mixture was vigorously shaken for 10 min and then allowed to stand for phase separation. The upper petroleum ether phase (containing residual lipids) was discarded. The lower ethanol phase was collected, transferred again to a rotary evaporator, and concentrated under reduced pressure at 50°C–60°C to a thick paste. After vacuum drying, the purified pomegranate peel polyphenol extract powder was obtained, which was sealed and stored away from light for future use.

### 2.3 Quantitative Analysis of EA and PU in Pomegranate Peel Polyphenol Extract via UFLC‐MS/MS

To determine the contents of the main polyphenolic components, EA and PU, in pomegranate peel polyphenol extract, an ultra‐fast liquid chromatography coupled with triple quadrupole mass spectrometry (UFLC‐MS/MS) method was employed. Sample preparation was performed as follows: accurately weighed 2.33 mg of pomegranate peel polyphenol extract powder was first dissolved in 20 μL of dimethyl sulfoxide (DMSO) with sonication, and then 2310 μL of 80% methanol–water solution was added. The mixture was subjected to three repeated ultrasonic extractions (20 min each). After centrifugation at 13,000 rpm for 10 min, the supernatant was collected as the sample for EA determination. An aliquot of the supernatant was further diluted three‐fold with 80% methanol–water for PU determination. For external standard calibration, stock solutions of EA and PU standards (25 mg/mL) were prepared in DMSO and then serially diluted with methanol to obtain calibration standards at concentrations of 100, 300, 1000, 3000, 10,000 ng/mL for EA and 100, 300, 1000, 3000, 20,000 ng/mL for PU.

Chromatographic separation was performed on an ACQUITY UPLC BEH C18 column (2.1 mm × 50 mm, 1.7 μm) at 35°C with a flow rate of 0.7 mL/min. The sample chamber temperature was maintained at 15°C, and the column equilibration time was 1 min. For EA analysis, the mobile phase consisted of 0.01% ammonia‐water (*v*/*v*) (phase A) and acetonitrile (phase B) with a gradient elution program of 0 min 90% A, 0.7 min 10% A, and 1.7 min 10% A. The injection volume was 2 μL. For PU analysis, the mobile phase consisted of 0.1% formic acid‐water (*v*/*v*) (phase A) and acetonitrile (phase B) with a gradient elution program of 0 min 90% A, 1.0 min 10% A, and 2.0 min 10% A. The injection volume was 1 μL. Mass spectrometric detection was performed using an electrospray ionization (ESI) source in negative ion mode with a capillary voltage of 5.5 kV and an ion source temperature of 500°C. Multiple reaction monitoring (MRM) parameters were set as follows: for EA, precursor ion m/z 301.2 → product ion m/z 284.1, declustering potential (DP) 100 V, collision energy (CE) −38 eV, dwell time 0.1 s; for PU, precursor ion m/z 1083.2 → product ion m/z 601.1, DP −220 V, CE −63 eV, dwell time 0.1 s. Data acquisition and processing were carried out using MultiQuant software (version 1.6.2) with external standard quantification, and Microsoft Excel 2021 was used for preliminary data processing.

### 2.4 Animal Experiments

All animal experiments were performed by the laboratory animal care and use guidelines. The animal handling procedures were also carried out in accordance with the “Guidelines for the Care and Use of Laboratory Animals at Shandong University of Technology”, and were approved by the Animal Ethics Committee of Shandong University of Technology (Approval Certification Number: YLX20220109). Six‐week‐old male specific‐pathogen‐free (SPF) grade Kunming mice were purchased from Shandong Laboratory Animal Center (Jinan, Shandong, China) (Approval Number: SCXK2022‐0013). The mice were kept in a standardizing laboratory maintained at a temperature of 25°C ± 3°C and a humidity of 55%–65% relative humidity on a standard 12 h light–dark cycle, with free access to water and food. Before starting the formal intervention, the mice underwent a 1‐week adaptive feeding period.

After 1 week of adaptation, all mice were randomly divided into 5 groups (*n* = 8): (I) a normal control group (NC), (II) a HUA group (HUA), (III) a low‐dose pomegranate peel polyphenol extract group (PPE‐L, 50 mg/kg), (IV) a high‐dose pomegranate peel polyphenol extract group (PPE‐H, 200 mg/kg), (V) a benzbromarone group (BEN, positive control, 20 mg/kg). Apart from mice in the NC group, all mice were given 5% fructose water and injected with 250 mg/kg of PO to induce HUA (B. Yang et al. 2023). PPE and benzbromarone were dissolved in 0.5% carboxymethylcellulose sodium and orally administered continuously for 4 weeks. One week before the experiment ended, feces and urine were collected and stored at −80°C. At the end of the experiment, the mice were euthanized by inhaling carbon dioxide. Blood was collected using heparin anticoagulant tubes and centrifuged at 3000 rpm for 10 min at 4°C for 10 min. The kidneys and other tissues were stored at −80°C.

### 2.5 Cell Culture and Intervention

Human proximal tubular epithelial cells (HK‐2) were cultured in DMEM/F12 medium (Meilun, Dalian, China) supplemented with 10% fetal bovine serum (FBS; Sinopharm, Beijing, China) and 1% penicillin/streptomycin at 37°C in a 5% CO_2_ atmosphere. Based on previous findings, after incubation with PPE or EA, the cells were stimulated with 8 mg/dL soluble UA solution to induce a cellular model of HUA. The safe concentrations of PPE and EA for in vitro administration were determined using the Cell Counting Kit‐8 (CCK‐8) assay.

### 2.6 Biochemical Measurement

Biochemical analysis of serum, urine, and feces was conducted using commercial kits (Nanjing Jiancheng Technology Co. Ltd., Nanjing, China) to estimate the following parameters: serum UA, urine UA, fecal UA, creatinine (CRE), and blood urea nitrogen (BUN) levels. The estimated glomerular filtration rate (eGFR) was also determined. An appropriate volume of liver homogenate was used to assess XOD activity. A microplate reader (Model Varioskan Flash) was purchased from Thermo Fisher Scientific Inc.

### 2.7 Histological Analysis

After sacrifice of the mice, part of the kidney tissue was collected from the mice in each group, cleaned, and fixed in 4% paraformaldehyde. Specimens were embedded in paraffin, and serial sections (4 μm thickness) were prepared. The sections were stained with hematoxylin and eosin (H&E) and Sirius red, and subsequently observed under a light microscope for pathological analysis. Sirius red staining was used for collagen determination, enabling quantitative morphometric measurements to be performed in locally defined tissue areas. Histopathological damage in the kidney was assessed based on H&E staining and scored from 0 (no injury) to 4 (severe injury) following a previously reported study (M.‐Q. Zhang, Sun, et al. 2023).

### 2.8 Western Blotting

Total proteins were extracted from HK‐2 cells and renal tissues using RIPA lysis and cytosolic protein extraction kits, respectively, according to the instructions of the manufacturer. Subsequently, cellular or tissue proteins were separated by SDS‐PAGE, and the resolved proteins were transferred onto PVDF membranes. These membranes were blocked with 5% non‐fat milk for 2 h, incubated with specific primary antibodies overnight, and then with appropriate secondary antibodies for another hour in sequence. The protein bands were exposed using an enhanced chemiluminescence (ECL) developing solution (Shenzhen Zhongkewei New Material Co. Ltd., Shenzhen, China) and quantified by ImageJ software. All protein expression levels were normalized to the internal reference β‐actin.

### 2.9 In Vitro XOD Inhibition Assay

The reaction mixture consisted of 130 μL of phosphate‐buffered saline (pH 7.5), 10 μL of an XOD solution (0.4 units/mL, Shanghai Yuanye Biotechnology Co. Ltd., Shanghai, China), and 10 μL of EA, PPE, or allopurinol solutions at various concentrations. In 96‐well plates, the reaction mixtures were incubated for 15 min at 25°C. Then, at 25°C, 50 μL of a 0.5 mM xanthine solution (Shanghai Yuanye Biotechnology Co. Ltd., Shanghai, China) in phosphate‐buffered saline was added to initiate the reaction for 10 min. Three replicates were performed for each sample. Xanthine is catalyzed by XOD to produce UA. By measuring the content of UA at 295 nm using a microplate reader (Model Varioskan Flash), we were able to determine the activity of XOD (Y. Zhang, Li, et al. 2023). The percentage of XOD inhibition was calculated using the following equation:

$$\text{Inhibition rate}\;\left(\%\right)=\frac{\left(A-B\right)-\left(C-D\right)}{\left(A-B\right)}\times 100$$

A: XOD activity without the test solution; B: the sample without XOD or the test solution; C: includes both XOD and the test solution; D: the test solution without XOD.

### 2.10 Screening of HUA Differential Genes Using the GEO and GeneCards Database

We conducted a systematic search of the GEO database (https://www.ncbi.nlm.nih.gov/geo/) and the GeneCards database (https://www.genecards.org/) using the keyword “HUA”. The GSE198133 dataset was selected for analysis, and differential gene expression analysis was performed using GEO2R (https://www.ncbi.nlm.nih.gov/geo/geo2r/). The sets of differentially expressed genes (DEGs) related to HUA from both databases were merged, and after removing duplicates, the related disease targets for HUA were obtained.

### 2.11 Screening of Targets for Bioactive Components (PU, EA) From PPE

Firstly, the structures of PU and EA were retrieved from the PubChem database. Secondly, these structures were imported into the SEA Search Server, SwissTargetPrediction, and SuperPred databases to retrieve or predict drug targets. Meanwhile, therapeutic targets for EA and PU were directly retrieved from the TCMSP database. Subsequently, these targets were merged and duplicates were removed to obtain the targets of the bioactive components for HUA treatment. Finally, a Venn diagram was generated using Venny 2.1.0 (https://bioinfogp.cnb.csic.es/tools/venny/) to visualize the intersection between the component targets and HUA‐related disease targets.

#### 2.11.1 PPI Network Construction

All of the intersection targets were analyzed using the STRING database (https://string‐db.org/) with the species restricted to *Homo sapiens* . The results were visualized and analyzed in Cytoscape (Version 3.9.1). The CytoHubba tool was employed to screen hub targets based on degree centrality.

### 2.12 GO and KEGG Enrichment Analysis

Functional enrichment analysis of the intersection targets was performed using the Metascape database (https://metascape.org/gp/). GO and KEGG pathway enrichment analyses were conducted with a significance threshold of *p* < 0.05, and the top 10 significantly enriched pathways were visualized. GO enrichment analysis includes cellular component (CC), molecular function (MF), and biological process (BP).

### 2.13 Statistical Analysis

Statistical analyses were performed using GraphPad Prism 8 software. All data are presented as the mean ± standard error of the mean (SEM). Comparisons among multiple groups were analyzed using one‐way analysis of variance (ANOVA) followed by Tukey's post hoc test. *p*‐values < 0.05 were considered statistically significant. In the figures, different letters (a, b, c, d, e) indicate significant differences between groups *p* < 0.05, while groups sharing the same letter denote no statistically significant difference *p* ≥ 0.05.

## 3 Results

### 3.1 Quantitative Analysis of EA and PU in PPE via UFLC‐MS/MS

**FIGURE 1:**
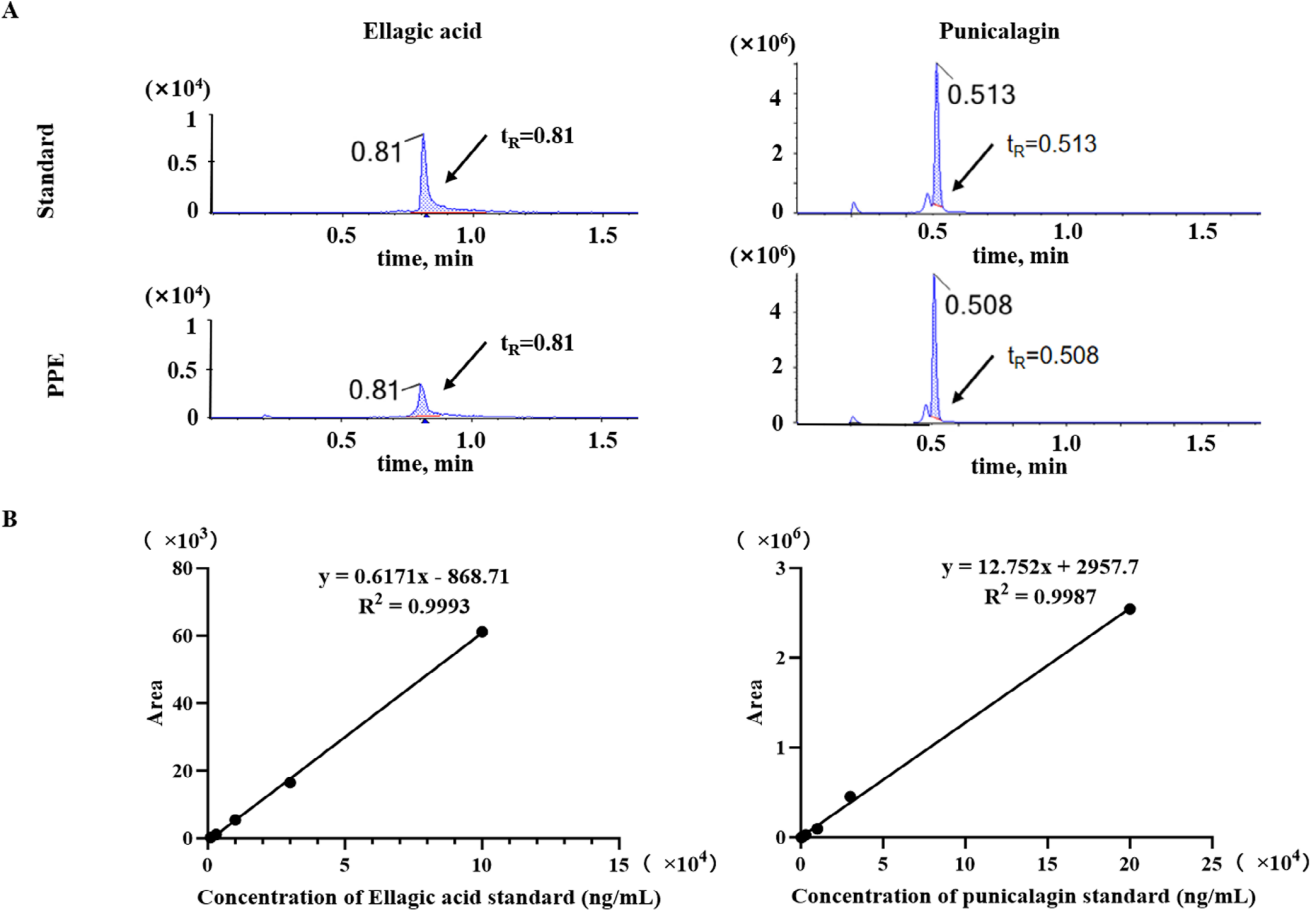
UFLC‐MS/MS analysis of EA and PU in PPE. (A) Representative MRM chromatograms of EA and PU standards (upper panels) and PPE samples (lower panels); (B) Standard calibration curves of EA and PU showing excellent linearity (*R* ^2^ > 0.99). EA, ellagic acid; MRM, multiple reaction monitoring; PPE, pomegranate peel extract; PU, punicalagin; UFLC‐MS/MS, ultra‐fast liquid chromatography–tandem mass spectrometry.

In this study, 50 g of pomegranate peel was used as raw material, and 9.13 g of PPE was obtained after extraction and purification, yielding an extraction rate of 18.26%. Since EA and PU have been reported as the main polyphenolic components in PPE, their contents were quantified by UFLC‐MS/MS. The UFLC‐MS/MS method was employed to quantify EA and PU in PPE. Under negative ion MRM mode, characteristic chromatographic peaks for EA were detected at a retention time of approximately 0.81 min in both the standard and the sample, while PU showed peaks at approximately 0.51 min (Figure 1A). Based on external standard calibration curves, which showed excellent linearity (*R* ^2^ > 0.99) over the tested concentration ranges (Figure 1B), the concentration of EA in the PPE solution (1 mg/mL) was determined to be 12709.43575 ng/mL, corresponding to a content of 3.81% (*w*/*w*) in the extract powder. For PU, the concentration in the three‐fold diluted extract solution was 88695.28701 ng/mL, which was calculated to be 0.088695287 mg/mL in the original extract solution (1 mg/mL), that is, 26.6% (*w*/*w*) in the extract powder.

### 3.2 PPE Reduces SUA Levels and Alleviates Renal Injury in HUA Mice

**FIGURE 2:**
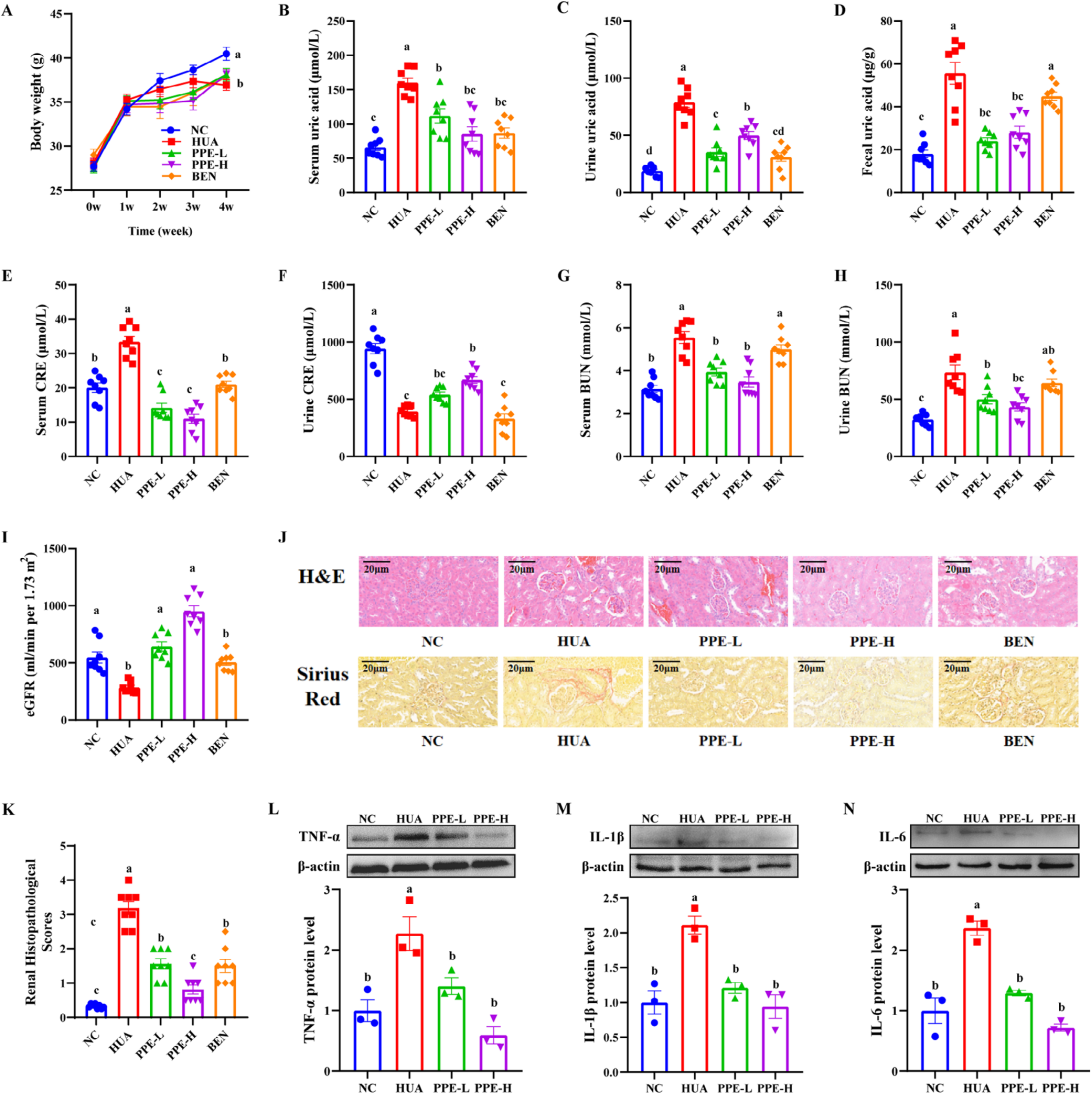
Effects of PPE on UA levels and renal function and inflammation in HUA mice. (A) Weekly body weight of each group of mice; (B) SUA level; (C) UUA level; (D) FUA level; (E) Serum CRE; (F) Urine CRE; (G) Serum BUN; (H) Urine BUN; (I) EGFR; (J) H&E and SiriusRed; (K) Semi‐quantitative histopathological scores of renal tubular injury; Western blots of TNF‐α (L), IL‐1β (M), and IL‐6 (N), and their relative expression levels. BEN, benzbromarone group; BUN, blood urea nitrogen; CRE, creatinine; eGFR, glomerular filtration rate; HUA, hyperuricemia group; NC, normal control group; PPE‐H, high‐dose pomegranate peel polyphenol extract group; PPE‐L, low‐dose pomegranate peel polyphenol extract group; XOD, xanthine oxidase. Scalebar is 20 μm. All data were analyzed by one‐way ANOVA, and Tukey's test was used for post hoc analysis; all data are presented as mean ± standard error (Means ± SEM). Letters (a, b, c, d) represent the results of multiple comparisons between different groups. The same letters indicate no significant difference (*p* ≥ 0.05), while different letters indicate significant differences in the statistics (*p* < 0.05).

To evaluate the effect of PPE on HUA, a mouse model of HUA was established. Compared with the control group, the body weight of mice in the HUA group was significantly reduced during the final week of the experiment (*p* < 0.05); although PPE intervention influenced body weight changes, the differences were not statistically significant (Figure 2A). Serum uric acid (SUA), urine uric acid (UUA), and fecal uric acid (FUA) levels in the HUA group were significantly higher than those in the control group (*p* < 0.05), indicating successful model establishment. Both low‐ and high‐dose PPE significantly reduced SUA levels in a dose‐dependent manner (*p* < 0.05), and the effect of high‐dose PPE was comparable to that of benzbromarone (Figure 2B). All three treatments also significantly decreased UUA levels (*p* < 0.05), and both low‐ and high‐dose PPE, as well as benzbromarone, significantly reduced UUA and FUA levels to varying extents, with PPE exhibiting a better effect on FUA improvement than benzbromarone (Figure 2C). Benzbromarone had no significant effect on fecal UA levels, whereas both low‐ and high‐dose PPE significantly reduced FUA levels (*p* < 0.05) (Figure 2D). These results indicate that PPE possesses potent UA‐lowering activity in vivo.

Compared with the control group, the HUA group exhibited significantly elevated serum creatinine (CRE) and blood urea nitrogen (BUN) levels, accompanied by decreased urine CRE and increased urine urea nitrogen levels (*p* < 0.05). These alterations were significantly reversed by treatment with low‐ and high‐dose PPE (*p* < 0.05), with no significant difference between the two doses, whereas benzbromarone only ameliorated the elevation of serum CRE (Figure 2E–H). Similarly, the estimated glomerular filtration rate (eGFR) was significantly decreased in the HUA group relative to the control group (*p* < 0.05), and this decrease was significantly ameliorated by low‐ and high‐dose PPE (*p* < 0.05), while benzbromarone treatment resulted in no significant change (Figure 2I). These findings demonstrate that PPE significantly attenuates renal injury and restores glomerular function (Han et al. 2025; B. Yang et al. 2023).

H&E (Hematoxylin–eosin staining) and Sirius red (Sirius red staining) staining were used to assess renal pathological injury and fibrosis. Kidneys in the HUA model group exhibited glomerular atrophy, tubular dilatation (H&E), and significant collagen deposition (Sirius red). Treatment with PPE (low/high dose) markedly attenuated these renal pathological abnormalities, including tubular injury and interstitial collagen deposition, and the ameliorative effect was more prominent in the PPE groups than in the BEN group (Figure 2J). Semi‐quantitative scoring further confirmed the renoprotective effect of PPE; the renal histopathological score was significantly higher in the HUA group than in the NC group, while PPE intervention dose‐dependently reduced the score, with the high‐dose PPE group showing a significantly lower score than the BEN group (Figure 2K). These results confirm that PPE alleviates HUA‐induced renal fibrosis and inflammation, consistent with the improvements in serum CRE and BUN levels.

As shown in Figure 2K–M, the protein expression levels of tumor necrosis factor‐α (TNF‐α), interleukin‐1β (IL‐1β), and interleukin‐6 (IL‐6) in the kidney were significantly higher in the HUA group than in the control group (*p* < 0.05). Treatment with PPE significantly suppressed the expression of these inflammatory cytokines in a dose‐dependent manner (*p* < 0.05), indicating that PPE alleviates HUA‐induced renal injury and inflammation.

### 3.3 PPE Inhibits XOD Activity and Modulates UA Transporter Expression

**FIGURE 3:**
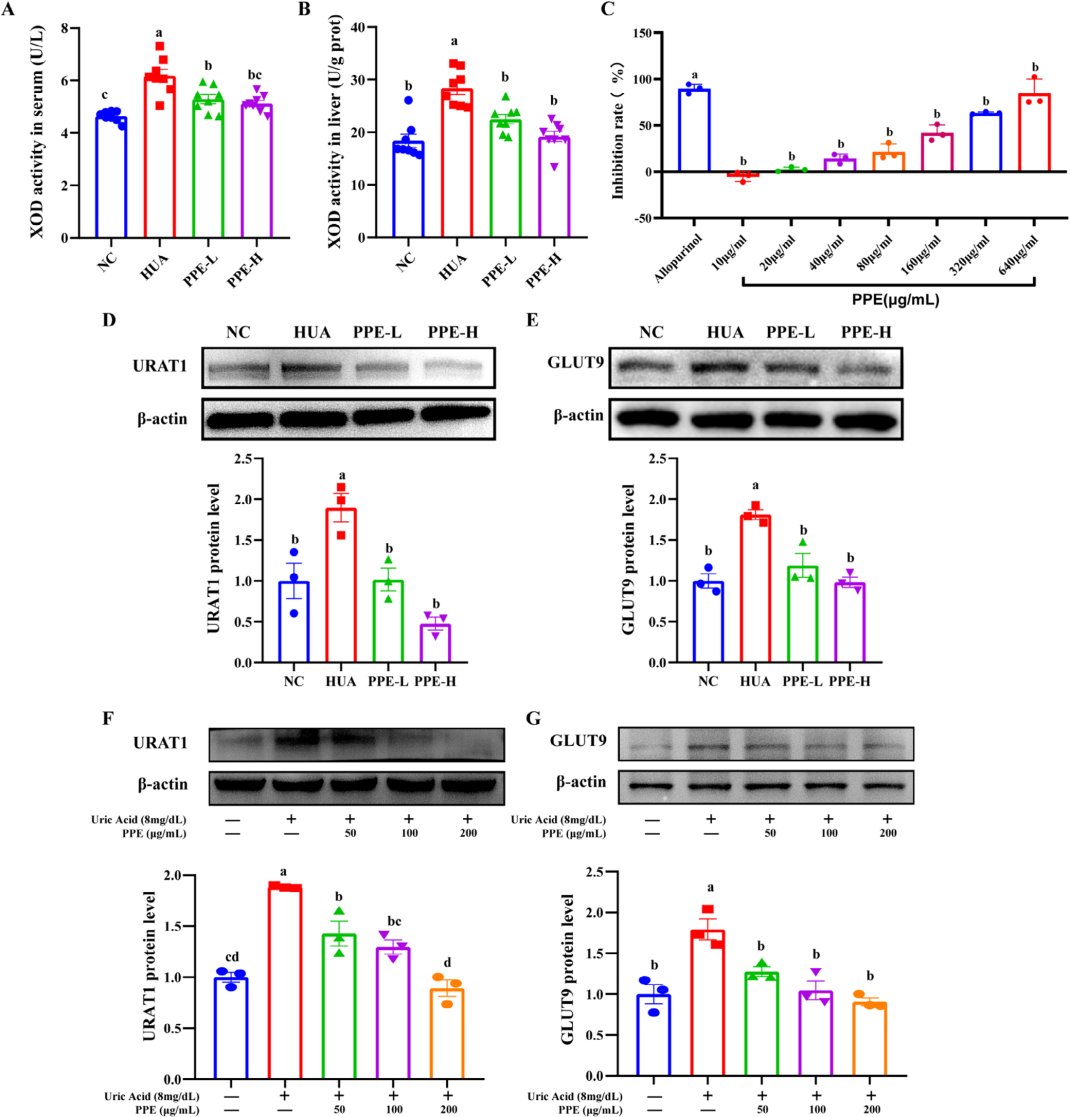
Effects of PPE on XOD and urate reabsorption transporters. (A) Blood XOD activity; (B) Liver XOD activity; (C) In vitro XOD activity inhibition rate of PPE; Western blots of urate transporter 1 (URAT1) (D) and glucose Transporter (GLUT9) (E) HUA mice kidneys, and their relative expression levels; Western blots of urate transporter 1 (URAT1) (F) and glucose Transporter (GLUT9) (G) in HK‐2 cells, and their relative expression levels. All data were analyzed using one‐way analysis of variance (One‐way ANOVA), and Tukey test was used for post hoc analysis; all were presented in the form of mean ± standard error (Means ± SEM). Letters (a, b, c, d) represent the results of multiple comparisons between different groups. The same letters indicate no significant difference (*p* ≥ 0.05), while different letters indicate significant differences in the statistics (*p* < 0.05).

To evaluate whether PPE affects UA production, XOD activity was measured because XOD is the rate‐limiting enzyme in purine metabolism (Ullah et al. 2024). Compared with the control group, plasma and hepatic XOD activities were significantly increased in HUA mice (*p* < 0.05). Following PPE treatment, XOD activity was significantly and dose‐dependently reduced (*p* < 0.05) (Figure 3A,B). The in vitro XOD inhibition assay further confirmed that PPE inhibited XOD activity in a dose‐dependent manner (Figure 3C). These findings indicated that PPE reduced UA production by inhibiting XOD activity, thereby exerting its UA‐lowering effect.

Approximately 90% of filtered UA is reabsorbed by renal proximal tubules, a process primarily mediated by the apical urate transporter URAT1 and the glucose transporter GLUT9 (Takata and Isomoto 2026; C. Wu et al. 2025). Western blot analysis revealed that, compared with the control group, the protein expression levels of URAT1 and GLUT9 in the kidneys of HUA mice were significantly upregulated (*p* < 0.05), parallel to impaired UA excretion. PPE administration dose‐dependently reversed these alterations in UA transporter expression (*p* < 0.05) (Figure 3D,E), suggesting that PPE reduces tubular UA reabsorption by inhibiting the expression of urate reabsorption transporters, thereby exerting a UA‐lowering effect.

The effect of PPE on the expression of UA transporters was further assessed in vitro using UA‐treated HK‐2 cells. As shown in Figure 3F,G, compared with the control group, the expression of URAT1 and GLUT9 was significantly upregulated in model cells (*p* < 0.05). Treatment with PPE concentration‐dependently reversed this dysregulation (*p* < 0.05), consistent with the in vivo findings.

### 3.4 PPE Ameliorates HUA by Inhibiting the Activation of PI3K‐AKT Phosphorylation

**FIGURE 4:**
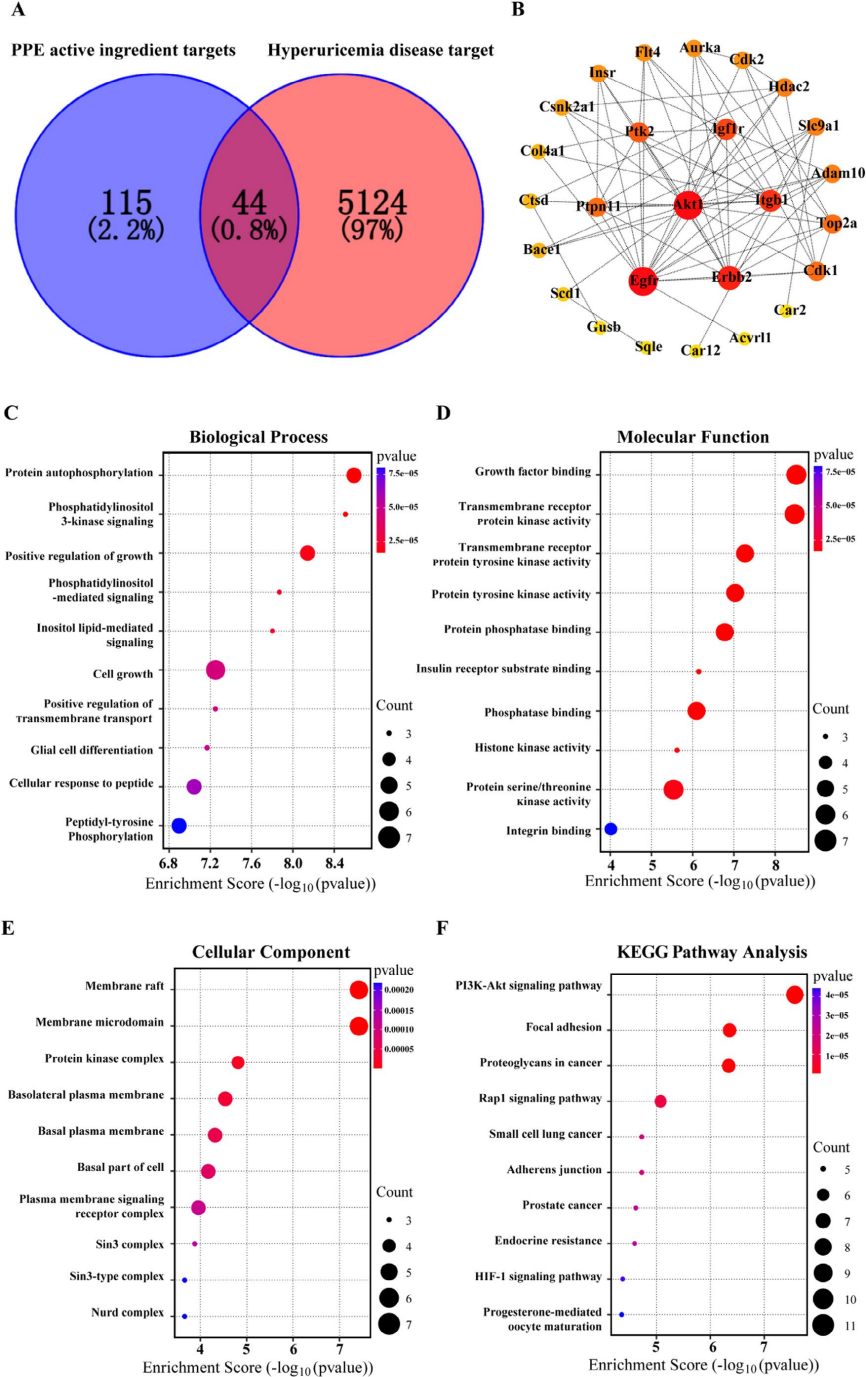
Network pharmacological analysis of the active components in pomegranate peel for the treatment of HUA. (A) Intersection of the active components of PPE and the disease targets of HUA in a Venn diagram analysis; (B) Protein interaction network diagram of the active components of pomegranate peel for treating hyperuricemia (in the diagram, the nodes represent target proteins, and the size of the nodes is positively correlated with the degree value; the lines represent the interaction relationships between the targets); (C) Bubble plot of GO enrichment of core targets in biological processes (BP); (D) Bubble plot of GO enrichment of core targets in molecular functions (MF); (E) Bubble plot of GO enrichment of core targets in cellular components (CC); (F) Bubble plot of KEGG enrichment pathways of core targets.

To explore the potential targets and pathways of PPE against HUA, network pharmacology analysis was performed. A total of 44 common targets between PPE and HUA were identified by Venn analysis (Figure 4A). The PPI network was constructed and analyzed using the STRING database and Cytoscape 3.9.1. Based on degree centrality, seven core targets were screened, including Akt1, Egfr, Erbb2, Itgb1, Igf1r, Ptk2, and Ptpn11 (Figure 4B). GO enrichment analysis indicated that the biological processes (BP) were mainly involved in protein autophosphorylation and phosphatidylinositol 3‐kinase (PI3K) signaling (Figure 4C). Molecular function (MF) analysis primarily involved growth factor binding and transmembrane receptor protein kinase activity (Figure 4D). Cellular component (CC) analysis mainly involved membrane raft, membrane microdomain, and protein kinase complex (Figure 4E). KEGG pathway enrichment analysis revealed that the ameliorating effect of PPE on HUA was potentially associated with the PI3K‐AKT signaling pathway (Figure 4F).

**FIGURE 5:**
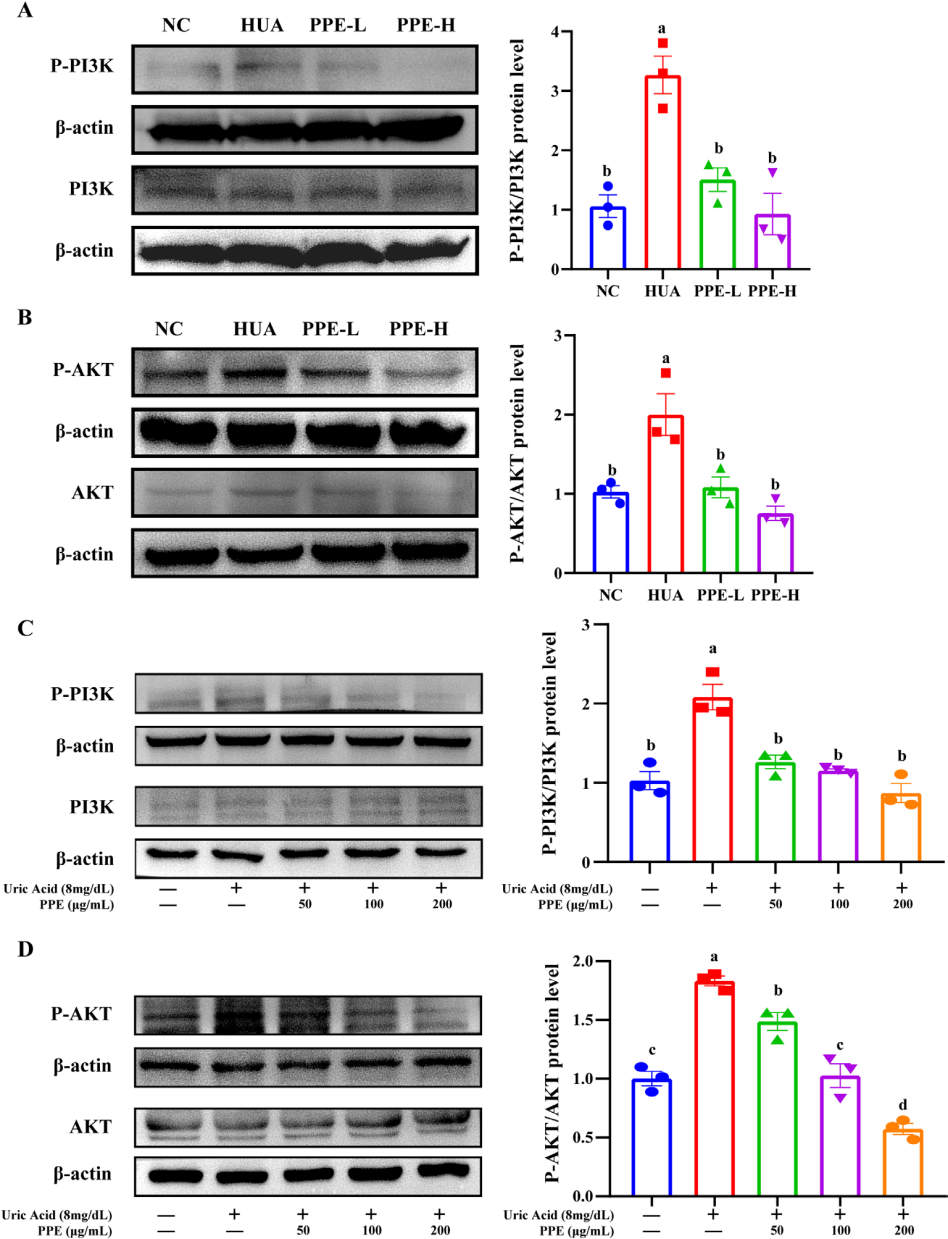
The effect of pomegranate PPE on the PI3K‐AKT pathway in hyperuricemic mice and UA‐induced HK‐2 cells. Western blots of p‐PI3K, PI3K (A), p‐AKT, and AKT (B), and their relative expression levels in HUA mice kidneys; Western blots of p‐PI3K, PI3K (C), p‐AKT, and AKT (D), and their relative expression levels in HK‐2 cells; All data were analyzed using one‐way analysis of variance (One‐way ANOVA), and Tukey test was used for post hoc analysis; all were presented in the form of mean ± standard error (Means ± SEM). Letters (a, b, c, d) represent the results of multiple comparisons among different groups. The same letters indicate no significant difference (*p* ≥ 0.05), while different letters indicate significant differences in the statistics (*p* < 0.05).

Given that KEGG analysis predicted the PI3K‐AKT pathway as a key pathway for PPE against HUA, its phosphorylation status was examined. Compared with the control group, the phosphorylation levels of PI3K and AKT in the kidney were significantly increased in HUA mice. PPE dose‐dependently reversed this elevation (Figure 5A,B). Consistent with the in vivo findings, PPE also significantly reversed UA‐induced PI3K‐AKT phosphorylation in HK‐2 cells (*p* < 0.05) (Figure 5C,D). These results indicated that PPE alleviated HUA by inhibiting PI3K‐AKT phosphorylation.

### 3.5 EA Modulated XOD Activity and UA Transporter Expression Through the PI3K‐AKT Signaling Pathway

**FIGURE 6:**
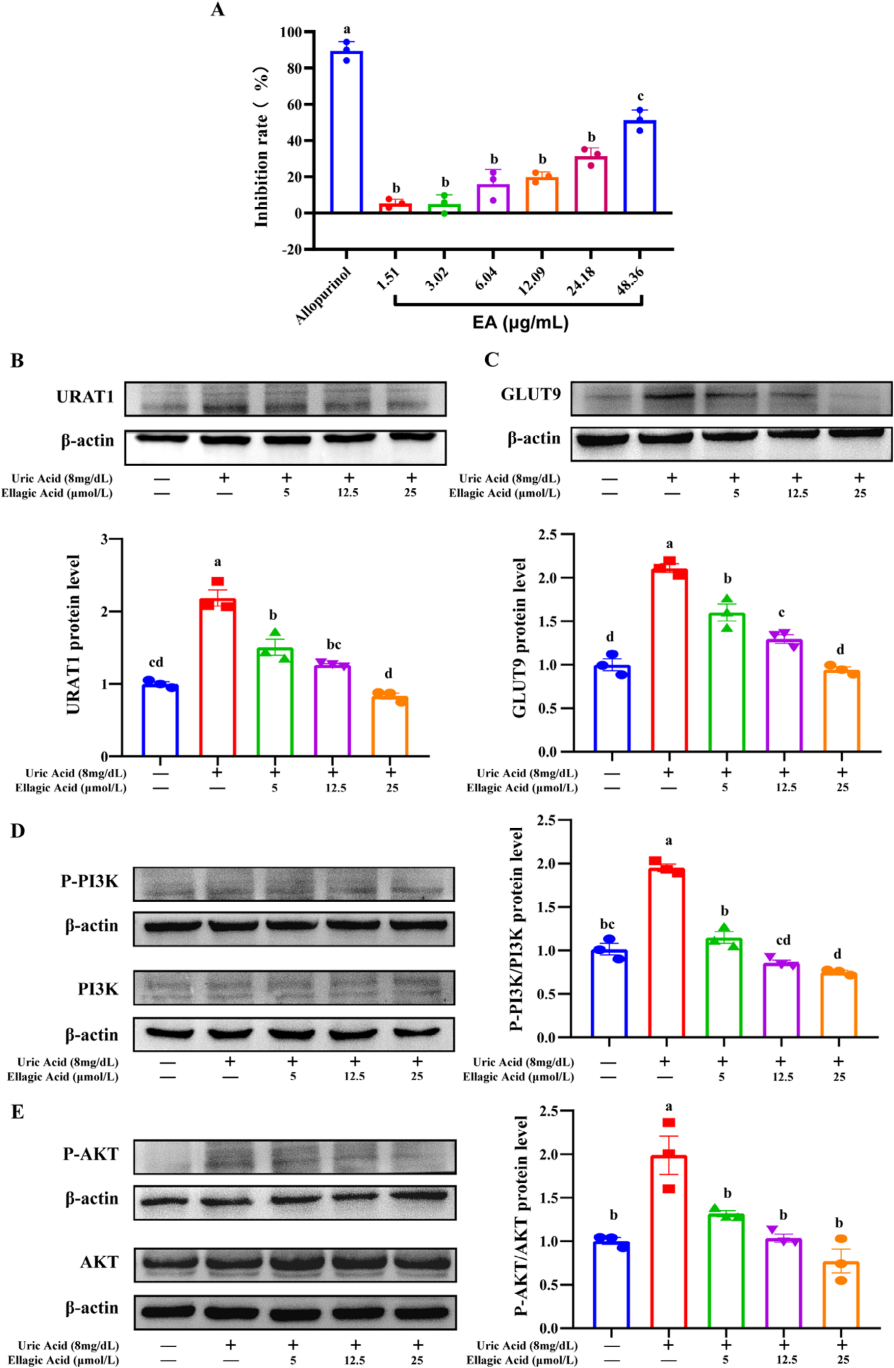
EA inhibits XOD activity in vitro and modulates the PI3K‐AKT pathway and urate transporters in UA‐induced HK‐2 cells. (A) In vitro XOD activity inhibition rate of ellagic acid (EA); Western blots of urate transporter 1 (URAT1) (B) and glucose transporter (GLUT9) (C), and their relative expression levels; Western blots of p‐PI3K, PI3K (D), p‐AKT, and AKT (E), and their relative expression levels in HK‐2 cells. All data were analyzed through one‐way analysis of variance (One‐way ANOVA), and Tukey test was used for post hoc analysis; all were presented in the form of mean ± standard error (Means ± SEM). Letters (a, b, c, d) represent the results of multiple comparisons among different groups, while different letters indicate significant differences in the statistics (*p* < 0.05).

Since PU, the most abundant component in PPE, can be hydrolyzed to EA in vivo, and EA as a small molecule with higher stability and bioavailability serves as a more direct acting entity, the effects of EA were further investigated (L. Du et al. 2018; Long et al. 2019; M. Zhang, Cui, et al. 2023). EA is the main characteristic component of PPE. The in vitro XOD inhibition assay demonstrated that EA inhibited XOD activity in a dose‐dependent manner (Figure 6A). In UA‐induced HK‐2 cells, the protein levels of the urate reabsorption transporters URAT1 and GLUT9 were significantly upregulated compared with the control group (*p* < 0.05). Treatment with EA significantly and dose‐dependently reversed this dysregulation (*p* < 0.05) (Figure 6B,C). Meanwhile, the phosphorylation levels of PI3K and AKT in the HUA group were significantly increased compared with the control group, which were also dose‐dependently suppressed by EA intervention (*p* < 0.05) (Figure 6D,E). These results suggested that EA inhibited XOD activity and downregulated URAT1 and GLUT9 expression by suppressing the activation of the PI3K‐AKT signaling pathway, which were consistent with the in vivo findings from PPE‐treated HUA mice, indicating that EA serves as the material basis for the anti‐hyperuricemic effect of PPE.

## 4 Discussion

HUA is a complex metabolic disorder resulting from purine metabolism dysregulation, characterized by an imbalance between UA overproduction and underexcretion. The present study demonstrated that PPE exerts potent UA‐lowering and renoprotective effects in a mouse model of HUA induced by potassium oxonate and fructose, as well as in UA‐stimulated HK‐2 cells. Mechanistically, PPE inhibited XOD activity to reduce UA synthesis and suppressed the PI3K‐AKT signaling pathway to downregulate urate reabsorption transporters URAT1 and GLUT9, thereby promoting renal UA excretion. These findings highlight PPE as a promising multi‐target candidate for HUA management.

XOD is the rate‐limiting enzyme in purine metabolism, and its inhibition is a classic strategy for lowering UA (Y. Li et al. 2025). Previous studies have shown that EA, a major active component of PPE, exerts antihyperuricemic effects by inhibiting hepatic XOD activity (J. Chen et al. 2023; Y.‐H. Wang, Peng, et al. 2025). Consistent with this report, our in vitro XOD inhibition assay confirmed that both PPE and EA dose‐dependently inhibited XOD activity. Importantly, beyond XOD inhibition, the present study further revealed that EA also downregulated URAT1 and GLUT9 expression via the PI3K‐AKT pathway, a mechanism that, to our knowledge, has not been previously reported for EA. Renal UA reabsorption is critically mediated by URAT1 and GLUT9 (Matsushita et al. 2025), and multiple studies have demonstrated that high UA levels activate PI3K‐AKT phosphorylation, leading to upregulation of these transporters (Fujii et al. 2025; Y. Zhang et al. 2021). Our results are in agreement with these observations, showing that PPE and EA reversed UA‐induced PI3K‐AKT activation and consequently reduced URAT1/GLUT9 expression. Additionally, PPE attenuated renal inflammation, as evidenced by reduced levels of TNF‐α, IL‐1β, and IL‐6 in HUA mouse kidneys. Accumulating evidence indicates that the cytokine‐epigenetic axis serves as a core regulatory module driving inflammatory injury in metabolic diseases, and natural bioactive compounds commonly exert anti‐inflammatory effects by targeting this axis (Luo et al. 2026). This anti‐inflammatory property may synergistically contribute to the renoprotective effects of PPE (J. Du et al. 2025).

Network pharmacology has become a valuable tool for predicting multi‐target mechanisms of herbal medicines (X. Li et al. 2023; P. Zhang, Zhang, et al. 2024; R. Zhang et al. 2019). Xu et al. identified 12 active components of Phellodendri Cortex and predicted their anti‐HUA effects via the PI3K‐AKT and NF‐κB pathways (Xu et al. 2022), which were subsequently validated by animal experiments. Zhang, Zhu, et al. (2024) demonstrated that berberine from *Portulaca oleracea* inhibited XOD and regulated urate transporters. In line with these studies, our integrated network pharmacology and GEO analysis identified seven core targets (Akt1, Egfr, Erbb2, Itgb1, Igf1r, Ptk2, Ptpn11) and pinpointed the PI3K‐AKT pathway as the key anti‐HUA mechanism of PPE. We prioritized validating this pathway for three main reasons. It exhibited the strongest statistical significance in KEGG enrichment, served as the topological hub of the target network with six of the seven core targets converging on it, and its activation is well documented to upregulate renal URAT1 and GLUT9, consistent with the core phenotype of impaired urate excretion in HUA. This strategy also follows the standard research paradigm for multi‐target natural products. The remaining six hub targets participate in HUA‐related renal fibrosis, matrix adhesion, metabolic dysregulation, and inflammation as upstream modulators of the PI3K‐AKT cascade, representing promising directions for further deciphering the full pharmacological network of PPE. These results confirm the network pharmacology prediction and support the PI3K‐AKT pathway as a critical mediator of the anti‐HUA effect of PPE.

However, several limitations of this study should be acknowledged. First, as a polyphenol extract, PPE may contain additional bioactive compounds beyond PU and EA that exert synergistic effects, warranting further investigation to clarify its pharmacodynamic material basis through spectrum‐effect relationship studies. Second, the molecular mechanism of EA's direct target engagement remains to be fully elucidated, and its specific role in regulating the PI3K‐AKT pathway requires further validation using techniques such as surface plasmon resonance (SPR) and gene silencing. Third, the potassium oxonate combined with fructose‐induced HUA mouse model used in this study mainly simulates secondary hyperuricemia caused by uricase inhibition and high fructose intake, which has certain differences from the pathological process of human primary hyperuricemia driven by polygenic inheritance and long‐term dietary factors. Future studies addressing these issues, combined with clinical samples, gene editing technologies, and multi‐omics analyses, are warranted to further elucidate the efficacy and mechanisms of PPE.

The present study demonstrated that PPE significantly reduced serum UA levels and ameliorated renal injury in potassium oxonate/fructose‐induced hyperuricemic mice, exhibiting favorable renoprotective effects. Network pharmacology combined with in vivo and in vitro experiments confirmed that PPE promoted renal UA excretion by inhibiting the PI3K‐AKT signaling pathway, and its key active component EA exerted consistent mechanistic effects in vitro to provide a more comprehensive scientific basis for its clinical application as a functional food or pharmaceutical agent.

## Author Contributions

**Pingping Guo:** validation, data curation. **Qingqing Han:** methodology, supervision, writing – review and editing, conceptualization. **Xinhua Song:** conceptualization, supervision, funding acquisition, resources. **Zeyu Yin:** software, investigation, formal analysis, data curation, visualization, writing – original draft. **Gangao Yang:** validation, data curation. **Shufei Liang:** software, data curation, investigation, formal analysis, visualization. **Chao Wang:** project administration, resources.

## Funding

This work was supported by the Natural Science Foundation of Shandong Province (Grant ZR2022MH306; Grant ZR2026MS1381).

## Conflicts of Interest

The authors declare no conflicts of interest.

## Data Availability

The data that support the findings of this study are available on request from the corresponding author. The data are not publicly available due to privacy or ethical restrictions.
